# Direct speciation analysis of thallium based on solid phase extraction and specific retention of a Tl(III) complex on alumina coated with sodium dodecyl sulfate

**DOI:** 10.1007/s00604-015-1624-3

**Published:** 2015-08-29

**Authors:** Ewa Biaduń, Monika Sadowska, Natalia Ospina-Alvarez, Beata Krasnodębska-Ostręga

**Affiliations:** Faculty of Chemistry, University of Warsaw, Pasteura 1, 02-093, Warsaw, Poland

**Keywords:** Thallium(III), Alumina, SDS, Solid phase extraction, ICP MS, Direct speciation analysis

## Abstract

Alumina (Al_2_O_3_) with an average particle size of 63 μm was modified with the anionic surfactant sodium dodecyl sulfate (SDS) and then applied to (i) solid phase extraction and separation of both thallium(I) and thallium(III), and (ii) preconcentration of Tl(III) from waste water samples. Only Tl(III), in the form of its complex with diethylenetriaminepentaacetate (DTPA), was retained on the sorbent, from where it can be eluted with 40 % nitric acid. Thallium species were then quantified by ICP MS. The method was characterized by a LOD of 25 pg of Tl(I) and 160 pg of Tl(III) in 10 mL samples. A large excesses of Tl(I) over Tl(III) was tolerated, and relatively high levels of other metal ions, such as a 500-fold excess of Pb(II) and Cd(II), and a 2000-fold excess of Zn(II), respectively, do not interfere. The sorbent was easily prepared and possesses a high loading capacity, and these properties make it an attractive material for rapid and efficient extraction and speciation of Tl.

**Graphical abstract:**
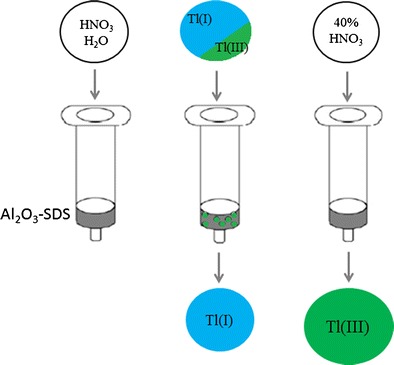
Schematic of the SPE procedure for separation (with preconcentration) of Tl(III) from Tl(I) was developed and applied to direct speciation analysis of thallium in wastewater. Self-made columns packed with alumina coated with SDS were used. The method is resistant to interferences from Pb, Cd, Zn and tolerates a large excess of Tl(I) over Tl(III).

Schematic of the SPE procedure for separation (with preconcentration) of Tl(III) from Tl(I) was developed and applied to direct speciation analysis of thallium in wastewater. Self-made columns packed with alumina coated with SDS were used. The method is resistant to interferences from Pb, Cd, Zn and tolerates a large excess of Tl(I) over Tl(III).

## Introduction

Thallium occurs in the environment at a low level of concentration; however, it is toxic even in trace amounts. The dominant species of Tl is a free cation of Tl(I), but also noticeable amounts of Tl(III) were found in water (21–26 ng L^−1^) [1], suspended particular matter of water (349 μg L^−1^) [2] and plants (110–230 μg kg^−1^) [3]. In organic form it was detected as dimethylthallium in the Atlantic Ocean (up to 3 ng L^−1^) [4]. All forms of Tl present in the environment have different toxicity. The toxicity of Tl(III) species highly depends on the chemical form. Therefore, speciation analysis of Tl is especially important. Thallium can easily penetrate into the body and replace potassium. Monovalent Tl also interacts with sulfur and sulfur contained in enzymes, which causes their deactivation. Thallium(III) is more toxic than Tl(I), Cu(II) and Cd(II), and similar to Hg(II) [5], as it causes creation of reactive oxygen species [6]. The solubility of Tl(I) compounds, except chlorides, is relatively high, and they can be transported by water to vast areas. Thallium can be accumulated in plant tissues and enter the food chain. Elevated levels of Tl in water pollute soil and plants and pose a threat to human health. Mobility of Tl in soil is a very important factor influencing the scale of toxic effects caused by this element [7]. Tl(I) is more stable in aqueous solutions while Tl(III) is more reactive and hydrolyses in neutral and alkaline solutions. Thallium(III) is less stable and can be electrochemically reduced to Tl(I) very fast (logK ≅ 40, E_red_(Tl^3+/^Tl^+^) = +1.26 V). The reduction is faster than complexation of Tl(III) (usually logK = 3÷9). At pH < 7 the hydrolysis of Tl(III) can be observed. In higher pH hydroxide complexes are formed. Reduction of Tl(III) to Tl(I) can be limited by complexation of Tl by reagents such as chlorides (logK = 17) [8], oxalates (logK = 16) [9], EDTA (logK = 37) or DTPA (logK = 46) [10]. The rate of reduction of Tl(III) in the presence of chlorides is up to 40 %, in the presence of acetates up to 80 % and in the presence of DTPA only 1–3 % of Tl(III) was reduced to Tl(I) [11]. However, the chelates of Tl(III) are not stable in the presence of plant matrix, and their reduction is accelerated by UV radiation. The stability constants of Tl(I) complexes with both inorganic and organic ligands are very low [12].

Chromatographic separation hyphenated with elemental detection is the most common method applied in speciation analysis. After conservation of the sample Tl(I) exists as a cation and Tl(III) as a large anion ([Tl(III)DTPA]^2−^ or Tl(III)Cl_4_^−^). Therefore, in the separation process cation exchange, anion exchange, and size exclusion mechanisms can be applied [8, 10, 13]. The separation can be carried out in the normal and reversed phase mode [10, 14]. The ratio between the content of Tl(I) and Tl(III) is a very important parameter determining the choice of chromatographic column.

The content of Tl(III) in environmental samples is at minor or even trace levels. It is difficult to determine without errors, particularly when the sample matrix is complex. Therefore, a possibility of separation and preconcentration of trivalent Tl is a crucial part of Tl speciation analysis. The extraction of Tl from water or extracts and absorption on a solid sorbent can be a good solution. In the case of Tl(III) the advantages of solid phase extraction are: separation of Tl(III) from dominating Tl(I) and interfering compounds, and preconcentration of Tl(III). SPE allows achieving Tl concentration above the determination limit. Moreover, the absorbed analyte can be stored for a long time. SPE is characterized by a high rate of recovery and it can be coupled with a various methods of determination, such as inductively coupled plasma mass spectrometry (ICP MS) [15], UV-Vis spectrophotometry [16] or atomic absorption spectrometry [17]. The differences in chemical properties of various forms of Tl are the basis of their separation and preconcentration from environmental samples. Trace amounts of Tl have been pre-concentrated on modified multi-layer carbon nanotubes [18]. Application of the positively charged nitrocellulose allows retaining Tl(III)Cl_4_^−^. The content of Tl(I) was determined indirectly [17]. Tl was also pre-concentrated from environmental samples on alumina modified with oxine. In the presence of EDTA only Tl(I) was retained. The total content was determined after reduction of Tl(III) [19]. Another sorbent applied for the analysis of water samples containing Tl(III) is silica gel with octadecyl-groups (SGX C18) chemically modified with DDTC. It forms complexes with both Tl(I) and Tl(III), but only Tl(III)DDTC is retained on the column. Before the analysis the samples were preserved – Tl(III) was converted into a complex Tl(III)DTPA [15]. SGX C18 was also used as a sorbent for complexes of TlCl_4_^−^ formed in water after addition of large amount of chlorides [20]. The chloride complex of Tl(III) was as well retained on the nano-alumina. Total content was determined after oxidation of Tl(I) to Tl(III) (indirect speciation) [16].

The aim of this study was to develop a simple analytical procedure of sample pretreatment – separation of Tl(I) from Tl(III)DTPA and preconcentration of Tl(III) from high sample volumes. Expected high enrichment should enable to determine traces of Tl(III) using elemental methods like anodic stripping voltammetry (ASV) and ICP MS. The study was based on the difference in retention of both Tl forms on alumina modified with an anionic surfactant - sodium dodecyl sulfate (SDS). The method was applied for quantitative determination of Tl(III) in wastewater collected in an industrial region of Southern Poland. Trace amounts of Tl(III) in this sample were already detected, and reported in our previous study [2].

## Experimental

### Chemicals

Sodium dodecyl sulfate (SDS) ReagentPlus ≥ 98.5 % (Sigma Aldrich, www.sigmaaldrich.com), Al_2_O_3_ (activity stage I) for column chromatography, particle size 0.063–0.200 mm (70–230 mesh ASTM) (Merck, www.merckgroup.com), acetone analytical grade (Chempur, www.chempur.pl), acetonitrile (ACN) gradient grade for liquid chromatography (Merck, www.merckgroup.com), ethanol 96 % (d = 0.808 g L^−1^) pure pro analysis (POCh, www.poch.com.pl), HNO_3_ 68 % (d = 1.42 g mL^−1^) ultranal (Cheman, www.ciechtrading.com), CH_3_COONa·3H_2_O (M = 136.08 g mol^−1^) pure pro analysis (Chempur, www.chempur.pl), CH_3_COOH 96 % (d = 1.06 g mL^−1^) Suprapur (Merck, www.merckgroup.com), diethylenetriaminepentaacetic acid (DTPA, M = 393.35 g mol^−1^) puriss pro analysis (Sigma Aldrich, www.sigmaaldrich.com), NaOH 30 % (d = 1.33 g mL^−1^) Suprapur (Merck, www.merckgroup.com), Tl(NO_3_)_3_· 3H_2_O (444.44 g mol^−1^) purum (Sigma Aldrich, www.sigmaaldrich.com). Standard solution of TlNO_3_ containing 1 mg L^−1^ Tl (d = 1.02 g mL^−1^) was obtained from Merck. All solutions were prepared using deionized water from Milli-Q-Water-System (Merck Millipore, USA, www.merckmillipore.com). The solution containing 0.15 mol L^−1^ DTPA was prepared by dissolving 5.9 g DTPA with 0.7 mL 30 % NaOH in water and diluting it to 100 mL. The 0.2 mol L^−1^ (pH 5.5) acetate buffer was prepared by dissolving 2.72 g CH_3_COONa· 3H_2_O and 220 μL 96 % CH_3_COOH in water, and diluting it to 100 mL. The standard solution of Tl(III) containing ca*.* 18 mg L^−1^ was prepared when needed by dissolving ca*.* 4 mg of Tl(NO_3_)_3_·3H_2_O (one crystal) in 100 mL of solution containing 0.025 mol L^−1^ DTPA and 0.1 mol L^−1^ acetate buffer (pH 5.5). Thallium(III) in water samples was stabilized by adding 1 mL of the 0.15 mol L^−1^ solution of DTPA to 5 mL of the water sample. 

### SPE procedure

The sorbent was prepared by mixing 500 g of alumina and 17 mL of solution containing 34 mg SDS [19]. Then the solution was stirred with magnetic stirrer for 10 min. The supernatant was decanted and the SDS-coated alumina was rinsed thoroughly with several portions of water. The wet sorbent was placed into a syringe (ϕ 13 mm) with a filter, packed using the vacuum pump, and covered with a second filter. To improve the homogeneity of the sorbent, it was loaded on the columns while still wet and then it was slowly dried.

Separation of the two forms of thallium [Tl(I) and Tl(III)DTPA] was performed using an Agilent SampliQ 12-Position Solid Phase Extraction chamber (Agilent, USA, www.agilent.com) connected with a vacuum pump (KNF Neuberger, Germany, www.knf.com) and handmade columns filled with modified alumina as a sorbent. Solution flow rate was 0.7 mL min^−1^. For separation and preconcentration of Tl(III) the following sequence of solutions was applied to the SPE column: (1) 2.0 mL of 0.1 mol L^−1^ HNO_3_, (2) 1.0 mL H_2_O, (3) 1–100 mL of sample, (3A) 3 mL of water (4) 3.0 mL of 40 % nitric acid. Steps 1 and 2 were applied to clean the column and to activate the active sites of the sorbent. Tl(I) is supposed to leave the column in step 3, while Tl(III)DTPA is retained in step 3 and then eluted in step 4. The scheme of the procedure is featured in Table 1.

**Table 1:** The SPE procedure for separation of Tl(I) from Tl(III) and preconcentration of Tl(III)

Step	Reagent	Aim
1	2.0 mL 0.1 mol dm^−3^ HNO_3_	Cleaning & Conditioning
2	1.0 mL H_2_O	pH increasing
3	5–100 mL of sample	Sample introduction [retention of Tl(III) and leaching of Tl(I)]
3A	3 mL H_2_O	Additional elution of co-retained Tl(I)
4	3 mL HNO_3_	Elution of Tl(III)

### Instrumentation

For spectrometric determination NEXION 300D mass spectrometer (Perkin Elmer, USA, www.perkinelmer.com) with Meihard-type nebulizer and Scott-type spray chamber was applied. ICP MS determinations were performed with the following parameters: sweeps: 40, replicates: 5, dwell time: 50 ms, ICP RF power: 1600 W, deflector voltage: -9 V, nebulizer gas flow: 1 L min^−1^, plasma gas flow: 18 L min^−1^, auxiliary gas flow: 1.2 L min^−1^ and monitored isotopes: ^203^Tl and ^205^Tl. Quantitative analysis program was used to automatically correct the intensities of interfering isobaric and molecular ions. For quantitative determinations the calibration curve method was applied.

The results obtained using ICP MS were compared with the results obtained using stripping voltammetry (ASV). The measurements were done using μAUTOLAB TYPE II (ECOCHEMIE, BV, The Netherlands, www.metrohm-autolab.com) with three electrodes: working electrode – hanging mercury drop electrode (HMDE), reference electrode – Ag/AgCl (3 mol L^−1^ KCl), auxiliary electrode – Pt wire. All measurements were performed in the range of potentials from −0.8 to 0.25 V. Tl signal was recorded at a potential −0.528 V. Preconcentration was carried out for 300–1000 s, deposition time was selected based on the content of Tl in the sample. Pulse amplitude was 25 mV s^−1^, and the polarization rate 20 mV s^−1^. For total Tl determination the applied electrolyte contained 9.9 mL of distilled water and 100 μL of conc. HNO_3_. To transform Tl compounds into free Tl^+^ ions the sample was evaporated to dryness with 1 mL conc. HNO_3_. The content of TI(I) in the presence of Tl(III)DTPA was measured in an electrolyte consisting of 1 mL of 0.2 mol L^−1^ acetate buffer, pH 5.5, 1 mL of 0.15 mol L^−1^ DTPA solution and 8 mL of DI water. For quantitative determinations the double standard addition method was used.

## Results and discussion

Thallium (III) is unstable in a solution, and undergoes a rapid reduction to Tl(I). To prepare a Tl(III) standard solution it is necessary to stabilize the trivalent form by addition of complexing agents. The most popular are chlorides and DTPA. If the concentration of Tl(III) is relatively high (some 10 mg L^−1^), Tl(Cl)_4_^−^ is stable enough. In solutions with low concentrations (below mg L^−1^ level) the reduction of Tl(III) is noticed (25–30 %) [10]. In such cases the addition of DTPA is required [8], but even then the reduction occurs, although it is significantly limited (3–5 %) [10, 13]. Tl(III)DTPA complex is very stable, in diluted solutions and also in the presence of other ions. The stability of this complex is practically independent of pH (the optimal pH range is 6–8). In our studies this ligand was used to stabilize Tl(III) in solutions.

### Retention of Tl standards on pure alumina

In view of the amphoteric character of alumina, it can be applied both as cation and anion exchanger, depending on the pH [21]. The preliminary study was done based on standard solutions. The solution containing 1.0 mL of 500 ng of TI(I) or 550 ng of TI(III) (as Tl(III)DTPA) was loaded on the SPE column (Al_2_O_3_). The effluent obtained in step 3 contained 8–11 % (*n* = 4) of added Tl(I) and the eluate obtained in step 4 contained only 0.1–0.2 % (*n* = 4) of added Tl(III). Both forms of Tl were retained on the sorbent.

Only Tl(I) ions can be determined by voltammetry, because only this species is accumulated on the electrode. Tl(III) bound in a complex compound is electrochemically inactive. The recovery of Tl introduced as Tl(III)DTPA was close to 0.2 % (*n* = 4) for the effluent obtained in step 3. Mineralization of the sample was applied to transform Tl compounds into free ions (the sample of effluent was evaporated to dryness with 1 mL conc. HNO_3_). For mineralized effluent, the recovery was slightly higher, close to 0.4 % (*n* = 4). The comparison of the results of ASV determination of Tl in effluent (step 3) prepared with and without the mineralization step, let to conclude that none of Tl forms left the column. Almost total retention of Tl(III) was observed.

Next, the composition of the eluent for thallium adsorbed as Tl(III)DTPA was optimized. According to the literature data, alumina is applied as a cation exchanger at pH about 8–9, and as an anion exchanger at pH 6–7. Usually to regenerate alumina sorbent strong alkaline solutions are used. Therefore, the solutions of NaOH (pH 7.3; 8.4; 10.5 and 11.5) were used to leach Tl species retained on the sorbent. However, the recovery study did not prove the theory, and the leaching efficiency did not exceed 0.4 %. Next, the solution of extremely low pH was checked as an eluate. In strongly acidic media the sorption capacity of alumina is low [21]. Solutions of HNO_3_ (pH 2.0 and 0) were checked. Only 60 % HNO_3_ allowed to leach 9.4–10.4 % of added Tl (*n* = 4). Such low recovery can be explained with the fact that anion-exchange properties change gradually over fairly broad pH ranges. Additionally, the elution with concentrated nitric acid caused some destruction of the sorbent.

### Retention of Tl on alumina modified with SDS

The literature data [16, 19] indicated that alumina modified with a surfactant can be a good sorbent to retain the studied analyte. The column with the sorbent was prepared according to the scheme presented below (see Analytical Application section). A recovery study was carried out with 1.0 mL of standard solution containing 200 ng of Tl(I), or 200 ng of Tl(III) loaded on SPE columns - Al_2_O_3_-SDS. The recovery of Tl(I) in the column effluent collected in step 3 was about 71–77 % (*n* = 4). The introduction of step 3A to wash Tl(I) adsorbed in step 3, increased the recovery of Tl(I) to 101–105 %. First attempt to elute the retained Tl(III) (step 4) was by using EtOH but the leaching of Tl(III) was close to 0 %. Next, 1 mL of pure ACN and 1 mL of 1 mol L^−1^ HNO_3_ were tested as eluents. The extractability of Tl(III) using ACN was very low, only 2–7 % of added Tl(III) was recovered. Elution with 1 mol L^−1^ HNO_3_ (pH 0) was more efficient – the recovery for Tl(III) was up to 15 %. In pH < 1 the stability of Tl(III)DTPA was extremely low, due to competitive reactions – strong protonation of DTPA and acceleration of Tl(III) reduction to Tl(I). It causes the elution of Tl retained as complex. Therefore, 10 mol L^−1^ (40 %) nitric acid was checked. The obtained results were satisfying, the recovery of Tl(I) and Tl(III) was close to 99 % (Table 2). The final procedure consisted of four steps (Table 1) and in further study such scheme was applied.

**Table 2:** Recovery [%] of Tl(I) and Tl(III) from samples with different excess of Tl(I) over Tl(III). The samples contained 20 ng Tl(III) and an excess of Tl(I) of 5, 10, 50, 100 and 200-fold in 10 mL. The results are presented as a mean value ± SD (*n* ≥ 3)

Step	Eluted Tl species	Tl(I):Tl(III) (contribution to the total Tl amount)						
		100 % : 0 %	0 % : 100 %	83 % : 17 %	91 % : 9 %	98 % : 2 %	99 % : 1 %	99.9 % : 0,1 %
				5 : 1	10 : 1	50 : 1	100 : 1	200 : 1
3	Tl(I)	96 ± 2 %	2.0 ± 0.6 %	81 ± 2 %	86 ± 2 %	96 ± 2 %	90 ± 1 %	41 ± 0.2 %
3A		3.0 ± 0.4 %	1.0 ± 0.1 %	2.0 ± 0.1 %	3.0 ± 0.3 %	4.0 ± 0.3 %	8 ± 1 %	26 ± 0.1 %
4	Tl(III)	< 0,1 %	96 ± 1 %	16 ± 3 %	10 ± 1 %	1.0 ± 0.5 %	1.0 ± 0.2 %	22 ± 0.2 %
	Total Tl	99 ± 2 %	99 ± 1 %	99 ± 4 %	99 ± 2 %	101 ± 2 %	99 ± 1 %	89 ± 1 %

The preparation of the SPE column was simple and inexpensive, as only one chemical modifier (SDS) is used. Additionally, the amount of sorbent packed into one column was only 500 mg (considerably less than in Dadfarnia et al. [19]). SDS, which is an anionic surfactant, forms micelles and retains Tl(III)DTPA^2−^ by formation of van der Waals forces between the nonpolar part of SDS and DTPA [21]. This method of extraction seems to be an effective and simple method of separation of Tl(III)DTPA^2−^.

### Sample volume

The study of sample volume is an important part of speciation analysis because preconcentration is necessary. Usually a sorbent which was modified by simple rinsing with reagents has low tolerance for large sample volumes. To recognize the breakthrough volume, samples containing 200 ng of Tl(I) or Tl(III) (as Tl(III)DTPA) in 5, 10, 50, 70 and 100 mL of solution were introduced to SPE columns (Al_2_O_3_-SDS). The results of Tl determination in eluates obtained in all procedure steps indicated the following recovery of thallium species: 95 ± 2 % for Tl(I) and 95 ± 4 % for Tl(III) for 5.0 mL sample, 99 ± 2 % for Tl(I) and 99 ± 1 % for Tl(III) for 10.0 mL sample, 98 ± 1 % for Tl(I) and 98 ± 3 % for Tl(III) for 50 ml sample, 97 ± 3 % for Tl(I) and 89 ± 3 % for Tl(III) for 70 mL sample, 95 ± 2 % for Tl(I) and 86 ± 2 % for Tl(III) for 100 mL sample. It was found that even 50–55 mL of sample can be applied. The 10 mL volume was selected for further experiments, because it was the most convenient to collect the eluates in 10 mL vials.

### Application of a mixture of Tl(I) and Tl(III)

The significant limitation in the speciation analysis of Tl is the large excesses of Tl(I) over Tl(III) in natural samples. During the following experiments various excesses of Tl(I) over Tl(III) were checked. The samples loaded on the SPE column contained Tl(III) (20 ng in 10 mL), and Tl(I) in 5, 10, 50, 100 and 200-fold excess (Table 2).

The recoveries of both Tl(I) and Tl(III) are ca. 100 % when the excess of Tl(I) is not higher than 200. When the excess was higher, total recovery decreased (to 89 %) and significant part of Tl was eluted in step 4.

Additionally, the capacity of the sorbent was evaluated. The samples applied to the SPE column contained Tl(III) (200, 400, 800 and 1000 ng in 10 mL). The recovery of Tl(III) was 0.1–0.2 % in step 3 and < 0.1 % in step 3A, indicating a very high capacity of the sorbent.

### Interferences

Thallium species are released into the environment mainly as a result of exploitation of sulfide ores [2]. Such ores contain small fractions of Tl and their main components include the ions of Zn, Cd or Pb [22]. These also react with DTPA and thus can compete with Tl in the adsorption process. Considering the above, interferences from various excesses of those metals were checked. The samples applied to the SPE column contained Tl(III) (10 ng in 10 mL, as Tl(III)DTPA) and 200, 500, and 700-fold molar excess of Pb(II) and Cd(II) and 500, 700, 2000-fold molar excess of Zn(II) (as nitrates) (Table 3). No interferences were caused by 500-fold excess of Pb and Cd, and 2000-fold excess of Zn (recovery ca. 100 %). Negative impact was only observed for 700-fold excess of Pb and Cd.

**Table 3:** Recoveries [%] of Tl in the presence of various interfering ions: Pb(II), Cd(II) and Zn(II) The samples contained Tl(III) (10 no in 10 mL, as Tl(III)DTPA), and 200, 500, and 700-fold molar excess of Pb(II) and Cd(II) and 500, 700, and 2000-fold molar excess of Zn(II). The results are presented as a mean value ± SD (*n* ≥ 3)

Step	Eluted Tl species	Pb			Cd			Zn		
		200	500	700	200	500	700	500	700	2000
3	Tl(I)	3.0 ± 0.4 %	3.0 ± 0.2 %	5 ± 3 %	3 ± 2 %	3 ± 1 %	7 ± 6 %	3.0 ± 0.6 %	3 ± 1 %	3 ± 1 %
3A		1.0 ± 0.4 %	1.0 ± 0.2 %	2.0 ± 0.4 %	0.6 ± 0.1 %	0.2 ± 0.1 %	1.0 ± 0.4 %	0.6 ± 0.4 %	0.6 ± 0.1 %	0.6 ± 0.1 %
4	Tl(III)	95 ± 4 %	94 ± 5 %	75 ± 7 %	100 ± 5 %	92 ± 2 %	74 ± 6 %	95 ± 1 %	94 ± 3 %	100 ± 3 %
	Total thallium	99 ± 4 %	98 ± 5 %	82 ± 7 %	104 ± 5 %	95 ± 2 %	82 ± 8 %	99 ± 1 %	98 ± 3 %	104 ± 3 %

### Analytical application

Wastewater samples were taken from a water outlet located in one of the most polluted areas in Poland (Upper Silesia). The wastes containing tailing minerals rich in Tl are deposited there in large ponds [23]. Samples of wastewater were taken in 1 L glass bottle, immediately acidified to pH 2 with HNO_3_ and stored until analysis. The original sample was slightly spiked with Tl(I) and Tl(III) standard solutions. The samples applied to the SPE column contained 40 ng Tl(III) and 10- or 50-fold excess of Tl(I) in 10 mL (Table 4). The recoveries of both Tl species were close to 100 %. The reproducibility of the procedure was about 2 % for Tl(I) and 1 % for Tl(III). Step 3A is necessary because of 3 % co-sorption of Tl(I) in step 3, and skipping step 3A can lead to wrong conclusions about Tl speciation.

**Table 4:** Recovery [%] of thallium from water samples spiked with Tl(I) and Tl(III). The samples contained 40 ng Tl(III) and 10 or 50-fold excess of Tl(I) in 10 mL. The results are presented as a mean value ± SD (*n* ≥ 3)

Step	Eluted Tl species	Tl(I):Tl(III) *	
		91 %:9 %	98 %:2 %
		10 : 1	50 : 1
3	Tl(I)	88 ± 2 %	96 ± 1 %
3A		3.0 ± 0.5 %	3.0 ± 0.8 %
4	Tl(III)	9.0 ± 0.5 %	2 ± 1 %
	Total thallium	100 ± 2 %	101 ± 1 %

*Contribution in the total Tl amount

The limit of detection (LOD) was calculated as a mean value increased by 3-times the standard deviation of Tl concentration in the blank sample (10 mL) (mean + $\overline{x}$ 3 SD, *n* = 4) and it amounted to 0.025 ng mL^−1^ for Tl(I) and 0.16 ng mL^−1^ for Tl(III). The limit of quantification (LOQ) was calculated as a mean value increased by 10-times the standard deviation of Tl concentration in the blank sample ($\overline{x}$mean + 10 SD, *n* = 4) and it amounted to 0.067 ng mL^−1^ for Tl(I) and 0.19 ng mL^−1^ for Tl(III).

A detailed comparison of this method to other methods of Tl(III) separation is presented in Table 5. The developed procedure allows determining Tl(III) below μg L^−1^ level in wastewater matrix. The LOD is lower than in methods described in the literature [15, 16, 19, 20]. The procedure is characterized by 50 mL breakthrough volume, which is equal to values reported for procedures based on retention on SGX C18 modified with a cationic surfactant [20] and slightly lower than for procedure based on Chromosorb105 [24]. It is also highly tolerant to the excess of Tl(I) over Tl(III). The tolerance is much higher than in methods mentioned above. The efficiency of retention and preconcentration is much less affected by the presence of Zn than it was mentioned in references [15, 16], and the retention is comparably affected by Pb. The great advantage of the procedure based on sorption on Al_2_O_3_-SDS is the possibility of direct determination of Tl(III) and Tl(I), without mathematical calculation of the concentration of one of Tl forms [15, 24]. Additionally, the preparation of a self-made sorbent significantly reduces the cost of analysis.

**Table 5:** Short comparison of methods of thallium speciation analysis using SPE for thallium species separation

Sorbent l	LOD [ng mL^−1^]	Effects of pH	Interfering ions (tolerable excess)	General advantages/disadvantages	Ref.
SGX C18 modified with DDTC	0.05 Tl(I) 0.21 Tl(III)	not studied	1000 - Cu(II), 700 - Zn(II) and Sn(II), 500 - Pb(II) and 100 - Cd(II)	direct speciation of Tl(I) and Tl (III)/low tolerance for Tl(I) excess	15
nano-Al_2_O_3_	800 Tl(III)	best sorption at pH 3.0–4.5	5 - Ca(II) and Mg(II), 10 - Zn(II), 1,5 Pb(II), 3,0 - Ga(III), 9,5 - Sb(III), 10 - Ge(IV), Se(IV), 7,5 - Re(IV), 2 Li(I), 5 V(V), 6 - Sr(II), 7 - Mo(VI), 10 - As(III), 70 - NO_3_^−^ and SO_4_^−^, 60 - CO_3_^2−^, 10 - PO_4_^3−^, 100 - Cl^−^	high sorption capacity and rapid separation/breakthrough volume lower than 25 mL and high LOD	16
Al_2_O_3_-oxine	2.5 Tl(I)	Tl(I) deposition at pH 2.5 to 11	1000 - Mg(II), Na(I) K(I)^,^ Zn(II), Pb(II), Cu(II), Co(II), Ni(II),Cs(I), I^−^, Cl, SO_3_^2−^; 750 - Cd(II), Ag(I), 500 - Ca(II), Fe(III), CO_3_^2−^	simple preparation of microcolumn, high reproducibility/long time of sample preparation before loading	19
modified silica	0.72 Tl(III)	not studied	250 - Cl^−^ and 100 - NO_3_^−^, SO_4_^2−^, Ca(II), Mg(II), K(I), Al(III) and Fe(III)	use of AAS and determination of Tl(III)/indirect speciation analysis of Tl	20
Chromosorb 105	0.034 Tl(III)	not studied	500 000-Ca(II) and Zn(II), 250 000-CO_3_^2−^, PO_4_^3−^, 5000 000- Fe(II), Fe(III), Al(III), Cl^-,^ 2500 000 Na(I) and K(I), 2000 000 Mg(II), 1000 000 SO_4_^2−^ and 25 000 Ni(II) and Cu(II)	simple, reliable way of Tl(III) preconcentration and low LOD/suitable only for AAS determination and important interferences from Cr and Co cations	24
Al_2_O_3_ - SDS	0.025 Tl(I) 0.16 Tl(III)	sample pH 5–6	500 - Pb and Cd, 2000 – Zn	direct speciation of Tl(I) and Tl(III), self-made column and high sorption capacity/use of conc. acid as eluent	this work

Abbreviations: SGX C18: silica gel with octadecyl-groups, DDTC: diethyldithiocarbamate, SDS: sodium dodecyl sulfate

## Concluding remarks

We developed a new method of direct speciation analysis of thallium, based on solid phase extraction using Al_2_O_3_-SDS as a sorbent. Only Tl(III) was retained. The procedure is highly tolerant to great excesses of Tl(I) over Tl(III), and to high content of interfering metals, such as Pb, Cd, and Zn. The great advantage of this method is the high capacity of the sorbent (up to 1 μg of Tl can be pre-concentrated on a single column). The method was successfully applied in case study analyzing wastewater samples. Low LOD, simple composition of the eluates and nearly no interfering ions enable its *offline* coupling with any of the elemental methods of detection, and make this method competitive to other methods previously described in the literature.
